# Oral and Palatal Dentition of Axolotl Arises From a Common Tooth-Competent Zone Along the Ecto-Endodermal Boundary

**DOI:** 10.3389/fcell.2020.622308

**Published:** 2021-01-11

**Authors:** Vladimír Soukup, Akira Tazaki, Yosuke Yamazaki, Anna Pospisilova, Hans-Henning Epperlein, Elly M. Tanaka, Robert Cerny

**Affiliations:** ^1^Department of Zoology, Faculty of Science, Charles University, Prague, Czechia; ^2^Max-Planck Institute of Molecular Cell Biology and Genetics (MPI-CBG), Dresden, Germany; ^3^Center for Regenerative Therapies, Technische Universität Dresden, Dresden, Germany; ^4^Department of Anatomy, Technische Universität Dresden, Dresden, Germany

**Keywords:** tooth development, initiation, patterning, dental arcades, ectoderm, endoderm, axolotl

## Abstract

Vertebrate dentitions arise at various places within the oropharyngeal cavity including the jaws, the palate, or the pharynx. These dentitions develop in a highly organized way, where new tooth germs are progressively added adjacent to the initiator center, the first tooth. At the same time, the places where dentitions develop house the contact zones between the outer ectoderm and the inner endoderm, and this colocalization has instigated various suggestions on the roles of germ layers for tooth initiation and development. Here, we study development of the axolotl dentition, which is a complex of five pairs of tooth fields arranged into the typically tetrapod outer and inner dental arcades. By tracking the expression patterns of odontogenic genes, we reason that teeth of both dental arcades originate from common tooth-competent zones, one present on the mouth roof and one on the mouth floor. Progressive compartmentalization of these zones and a simultaneous addition of new tooth germs distinct for each prospective tooth field subsequently control the final shape and composition of the axolotl dentition. Interestingly, by following the fate of the GFP-labeled oral ectoderm, we further show that, in three out of five tooth field pairs, the first tooth develops right at the ecto-endodermal boundary. Our results thus indicate that a single tooth-competent zone gives rise to both dental arcades of a complex tetrapod dentition. Further, we propose that the ecto-endodermal boundary running through this zone should be accounted for as a potential source of instruction factors instigating the onset of the odontogenic program.

## Introduction

One of the most important features of vertebrate dentitions is the patterned arrangement of their elemental units, the teeth. Each tooth has its defined position and a particular relation to other teeth of that dentition. In extant vertebrates, tooth development is restricted to the oropharyngeal region, where the locally thickened epithelium demarcates the prospective tooth-forming areas. This so-called odontogenic band usually invaginates into the underlying mesenchyme where it forms an epithelial strand, the dental lamina.

Shh and Pitx2 represent earliest markers of odontogenesis and factors responsible for tooth development in vertebrates. Absence of Shh or Pitx2 signaling results in the loss of tooth signaling centers, defects in tooth development, and missing teeth (Lin et al., 1999; Dassule et al., 2000; Cobourne et al., 2004; Jackman et al., 2010; Yu et al., 2020), and a lack of co-expression of *Shh* and *Pitx2*, for example, is associated with toothlessness in cypriniforms (Jackman et al., 2010). Once *Shh* and *Pitx2* specify the positions of the nascent dentition, a sequence of events leading to the appearance of the first tooth controls the addition of further tooth germs adjacent to this initiator-tooth (Gibert et al., 2019; Sadier et al., 2019, 2020). The way in which new tooth germs are sequentially added in the vicinity of this initiator-tooth then leads to the final appearance of the respective tooth field.

While the expression of *Shh* and *Pitx2* and the induction from the initiator-tooth may be among the earliest events of the developing dentition, it is not satisfactorily explained how the spatial distribution of this initial odontogenic potential within the oropharyngeal cavity is regulated (Balic, 2019). Several cues have been proposed including (1) the polarization and regionalization of the mandibular arch along the proximo-distal axis (Sharpe, 1995; Tucker and Sharpe, 2004), (2) activator/inhibitor interactions specifying tooth-competent regions (Sarkar et al., 2000; Fraser et al., 2008; Zhang et al., 2009), (3) the progressive and reiterated partitioning of the initially established tooth-competent region (Jernvall and Thesleff, 2000; Fraser et al., 2008), or (4) the influence of epithelial germ layers contributing to different parts of the oropharyngeal cavity (Smith, 2003; Ohazama et al., 2010).

Various roles have been assigned to the ectoderm and endoderm in triggering odontogenesis and patterning the teeth (Smith, 2003; Huysseune et al., 2009; Ohazama et al., 2010). However, a definite distinction of these germ layers fails due to the lack of general germ layer-specific morphological markers. The germ layer-specific expression of *Pitx2* (oral ectoderm) and *Shh* (endoderm) is present only until the rupture of the oral membrane (Helms et al., 1997; Schweickert et al., 2001; Eberhart et al., 2006). Therefore, uncovering the roles of these epithelia in tooth development requires an experimental germ layer-specific labeling, which, for now, is, to a large extent, pending.

**FIGURE 1:**
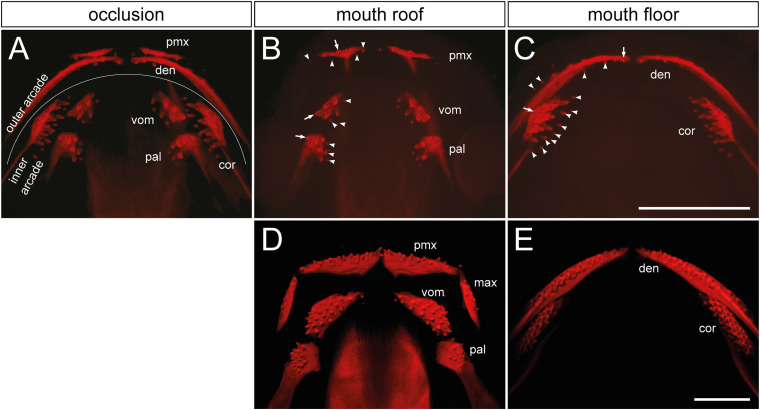
Distribution and composition of axolotl tooth fields. **(A)** The dentition of an axolotl larva (19 mm TL) is composed of several tooth fields, which are assembled into outer (premaxillary and dentary) and inner dental arcades (vomerine, palatine, and coronoid). These arcades show various field-specific arrangements. Outer arcade fields form initially a single tooth row; inner arcade fields constitute tooth patches. **(B,C)** Separation of mouth roof from floor shows presence of the oldest tooth (arrow) and addition of new tooth germs (arrowheads) within each field. **(B)** In the premaxillary field (pmx), new germs are added laterally and medially as the field stretches along the upper jaw, and new germs are also added posteriorly. Vomerine (vom) and palatine (pal) fields develop by addition of new germs medially from the oldest teeth located laterally. **(C)** In the dentary field (den), the oldest tooth is present close to the mandibular symphysis and new germs are added laterally along the lower jaw, thus forming the tooth row. New germs are further initiated lingually. In the coronoid (cor) field, the oldest tooth is located labially and new germs are added lingually in a hand fan-shaped manner. **(D,E)** The dentition of a 41 mm TL axolotl larva shows a single-rowed arrangement of premaxillary (pmx), maxillary (max), and dentary (den) fields, and a multi-rowed arrangement of vomerine (vom), palatine (pal), and coronoid (cor) fields. Scale bars equal 1 mm.

The Mexican axolotl is an extant amphibian with teeth assembled into dental arcades located in oral and palatal regions. The outer arcade is composed of single-rowed premaxillary, maxillary, and dentary tooth fields, and the inner arcade is composed of multi-rowed vomerine, palatine, and coronoid (sometimes called splenial) tooth fields (Figure 1). The composition of the axolotl dentition is typical for extant and extinct tetrapods (Matsumoto and Evans, 2017), yet how this complex dental system becomes established developmentally has not been addressed. The Mexican axolotl further provides an opportunity to experimentally assess the germ-layer origin of oral epithelia (Soukup et al., 2008) and thus, in combination with the information on the developmental origin of the respective tooth fields, represents an eligible developmental model for the understanding of the early events in the initiating dentition.

## Materials and Methods

### Handling of Axolotl Embryos and Transplantation of GFP-Oral Ectoderm

White mutant strain and white GFP-transgenic (Sobkow et al., 2006) axolotl embryos were acquired from the axolotl colony at MPI-CBG Dresden, Germany. Eggs prepared for prospective oral ectoderm transplantations were rinsed in tap water and put into sterile Steinberg solution containing antibiotics. Early neurulae (stage 14, Bordzilovskaya et al., 1989) were placed into a 2% agar-covered petri dish, manually decapsulated, and inserted firmly into deepenings cut into the agar. Oral ectoderm was transplanted from white GFP donors into white hosts at the early neurula stage (stage 14) as previously described (Soukup et al., 2008), and the larvae were fixed by hatching (stage 44) for whole mount analysis of the ectodermal extent in the oral cavity (*n* = 8). Further oral ectoderm transplantation experiments were performed for histological analysis of tooth addition at earlier stages. Embryos and larvae were anesthetized with MS-222 (Sigma) and fixed overnight in 4% paraformaldehyde in 0.1 M phosphate-buffered saline. Specimens prepared for *in situ* hybridization were dehydrated and stored in methanol at −20°C.

### *In situ* Hybridization

*In situ* hybridizations were performed on whole mounts using DIG-labeled RNA probes. Antisense probes for *in situ* hybridization were obtained by *in vitro* transcription using primers listed in Supplementary Table 1. Rehydrated larvae were digested in 20 μg/ml proteinase K, fixed in 4% paraformaldehyde for 10 min, transferred into hybridization solution (50% formamide, 4 × SSC, 0.1 mg/ml heparin, 1 × Denhardt’s, 0.1% CHAPS, 0.2 mg/ml yeast RNA, 10 mM EDTA, and 0.1% Tween-20) and incubated overnight in hybridization solution containing probe (1:1,000). Next day, the specimens were washed several times in post-hybridization solution (50% formamide, 4 × SSC, 0.1% Tween-20) and transferred via MABT buffer (100 mM maleic acid, 150 mM NaCl, 0.1% Tween-20) into blocking solution (2% blocking reagent, 20% sheep serum, in MABT). Following blocking, the specimens were incubated overnight in the blocking solution containing alkaline phosphatase-conjugated antibody against DIG (Roche, 1:3,000) at 4°C. The specimens were washed several times in MABT buffer, transferred into NTMT (0.1 M Tris, 0.1 M NaCl, 0.05 M MgCl_2_, and 0.1% Tween-20) and incubated in BM Purple substrate (Roche) until the desired signal developed. The following numbers of whole-mount hybridized specimens were analyzed: *n* = 20 (*Shh* mouth floor), *n* = 19 (*Shh* mouth roof), *n* = 39 (*Pitx2* mouth floor), *n* = 34 (*Pitx2* mouth roof).

### 3D Model of Axolotl Dentition Development

Axolotl larvae at stages 38, 41, and 42 were sectioned sagittally with a cryomicrotome at 10 μm thickness and processed through *in situ* hybridization with the *Pitx2* probe. Serial sections were photographed and used for computer-assisted 3D reconstruction. Alignment, segmentation, and surface rendering were carried out in Fiji using TrakEM2 plugin for ImageJ (Cardona et al., 2012; Schneider et al., 2012, Schindelin et al., 2012, 2015).

### Alizarin Red Staining

Axolotl larvae were bleached in a solution containing 3% H_2_O_2_ and 0.5% KOH (1:4) with the help of cold light and stained by a saturated solution of Alizarin red (Fluka) plus 0.5% KOH (1:16) overnight. Stained larvae were washed thoroughly by 0.5% KOH to remove excess stain and transferred slowly through a graded series of 0.5% KOH and glycerol (4:1, 2:1, 1:1, 1:2, 1:4) into 100% glycerol.

### Image Acquisition and Processing

Following *in situ* hybridization, larvae at stages 37–44 were dissected at the level of the jaw joint to enable whole mount visualization of teeth that had developed on the roof and floor of the mouth. Additionally, mouth roof epithelia of embryos hybridized with the *Shh* probe were excised and photographed as flat-mounts to prevent masking of the tooth-specific signal from the signal in the overlying neural tube. Whole mounts processed through *in situ* hybridization, skeletal stainings, and transplantation of GFP-labeled oral ectoderm were photographed as Z-stacks using motorized dissection microscopes (Olympus SZX12, Zeiss SteREO Lumar.V12). The final deep-focus images were acquired by merging the Z-stacks using maximum projection function. Larvae processed through transplantation of GFP-labeled oral ectoderm were further sectioned using the CM3050 cryomicrotome (Leica) and individual tooth germs were visualized using an anti-calbindin antibody (Sigma) as previously described (Barlow and Northcutt, 1997; Soukup et al., 2008). Z-stacks of sections were taken on the Olympus Cell^*R*^ IX81 microscope and merged as maximum projection images.

## Results

### The Complex Tetrapod Dentition of Axolotl Arises by Separation of Initially Compact Tooth-Competent Zones

**FIGURE 2:**
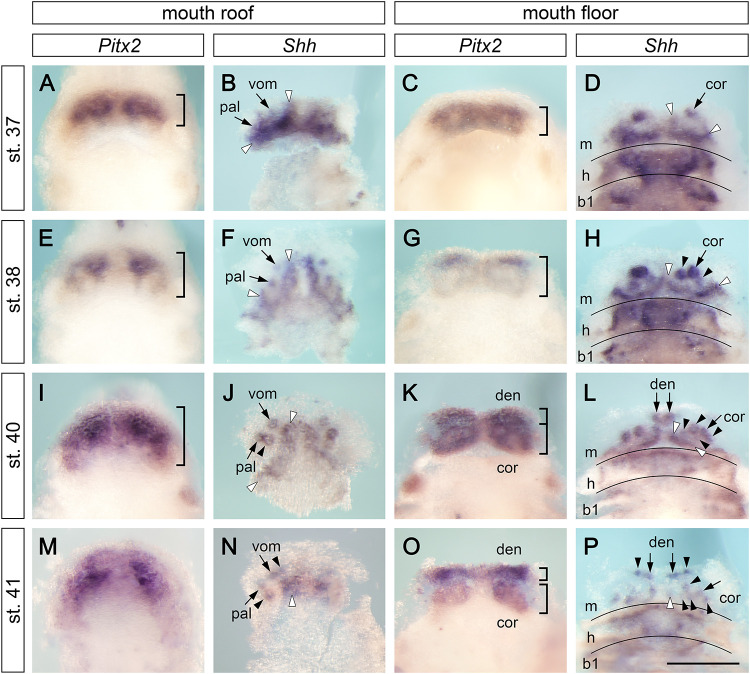
Expression patterns of *Pitx2* and *Shh* at early stages of axolotl odontogenesis. Expression patterns of early odontogenic markers *Pitx2* and *Shh* on the mouth roof and floor whole mounts show initial stages of development of axolotl dentition (in case of *Shh* expression, the epithelium of the mouth roof was dissected from whole mounts to circumvent masking of the tooth-restricted epithelial signal by the strong *Shh* signal from the adjacent neural tube). At stages 37–41, *Pitx2* labels tooth-competent regions while *Shh* is restricted to individual tooth germs and into postero-medially placed odontogenic bands (visualized as strands of *Shh* expression demarcated by white arrowheads). Positions of tooth germs are visible as regions of lower *Pitx2* expression. **(A,E,I,M)** Expression pattern of *Pitx2* on the mouth roof is initially restricted to its anterior part but becomes broader at later stages (parentheses). **(B,F,J,K)** Focal expression of *Shh* illustrates sequential addition of tooth germs starting from the initiator-teeth of vomerine (vom) and palatine (pal) teeth (**B,F**, arrows). New tooth germs are added postero-medially in the palatine tooth field (**J**, black arrowheads) and antero-medially in the vomerine tooth field (**N**, black arrowheads). **(C,G,K,O)** Expression pattern of *Pitx2* on the mouth floor enlarges posteriorly throughout development and, at stages 40–41, the pattern separates into the prospective dentary (den) and coronoid (cor) tooth fields (**K,O**, parentheses). **(D,H,L,P)** Focal *Shh* expression on the mouth floor marks positions of initiator-teeth of the coronoid (**D,H**, arrows) and dentary fields (**L,P**, arrows) and the addition of new tooth germs within both tooth fields (**H,L,P**, black arrowheads). New tooth germs are added lingually to the initiator tooth germ in the coronoid tooth field (**H,L,P**, black arrowheads) and laterally from the medially positioned initiator tooth in the dentary tooth field (**P**, arrowheads). b1, first branchial arch; cor, coronoid tooth field; den, dentary tooth field; h, hyoid arch; m, mandibular arch; pal, palatine tooth field; vom, vomerine tooth field. Scale bar equals 500 μm.

The outer and inner dental arcades of the axolotl dentition are initially composed of five, and later of six, pairs of tooth fields (Figure 1). In order to understand how this dentition acquires such a complex arrangement, we studied the expression of the early odontogenic markers *Pitx2* and *Shh*. At early larval stages (stages 37–41), *Pitx2* is expressed over the extents of the jaws and delineates tooth-competent zones. In contrast, *Shh* expression is focally restricted to initiating tooth germs and odontogenic bands, from where new germs will arise (Figure 2). *Pitx2* expression becomes later downregulated at sites where new tooth germs are added, a situation reminiscent of other vertebrates (Keränen et al., 1999; Fraser et al., 2004, 2008). At stage 37, *Pitx2* expression is restricted into broad domains on the roof and floor of the mouth (Figures 2A,C). The first tooth germs are initiated at this stage, as evidenced by the focal expression of *Shh*. They belong to the prospective vomerine, palatine, and coronoid tooth fields, i.e., constituents of the inner dental arcade (Figures 2B,D, small arrows). Additional strands of *Shh* expression are found on the roof and floor of the mouth labially from these first germs. They represent odontogenic bands from where new germs will be generated (Figures 2B,D, white arrowheads). During development, the *Pitx2*-expressing tooth-competent zones progressively extend posteriorly on both the roof and floor of the mouth (Figures 2A,C,E,G,I,K, parentheses) and, concomitantly, the *Shh*-expressing odontogenic bands align to the labial limits of these zones (Figures 2B,D,F,H,J,L). Based on the focal expression of *Shh* during subsequent stages, new tooth germs are added adjacent to the initiator-teeth, i.e., postero-medially in the palatine field (Figure 2J, arrowheads) and both postero-laterally and postero-medially in the coronoid field (Figure 2H, arrowheads). At stages 40 and 41, the splitting of the *Pitx2* expression pattern on the mouth floor suggests a separation of the tooth-competent zone into dentary and coronoid fields (Figures 2K,O). Meanwhile, the first tooth germs of the dentary tooth field are initiated at the mandibular symphysis (Figure 2L, small arrows). During subsequent stages, the *Shh* expression pattern labels newly initiated tooth germs that are added antero-medially in the vomerine tooth field (Figure 2N, arrowheads), laterally along the lower jaw in the dentary tooth field (Figure 2P, arrowheads) and postero-medially in the coronoid tooth field (Figures 2L,P, arrowheads). Further analysis of expression patterns of other odontogenic factors, such as *Bmp2*, *Bmp7*, or *Dlx5*, confirms the addition of new tooth germs within individual tooth fields, which are delineated by the expression of *Dlx3* (Supplementary Figure 1). Moreover, focal expression of *Bmp2*, *Bmp7*, and *Dlx5* labels the initiation of the first tooth germ of the premaxillary field at stage 41 (Supplementary Figures 1A,C,G, arrow). In order to further analyze the progressive compartmentalization of tooth-competent zones (Figure 2), we performed an independent analysis using 3D reconstructions of the roof and floor epithelia of the axolotl mouth based on serial sections hybridized with the *Pitx2* probe (Supplementary Figure 2). Concordant with data from whole mounts, stage 38 shows a compact expression pattern in the basal epithelial layer of the roof and floor epithelia of the prospective mouth. At subsequent stages, the common *Pitx2* expression pattern becomes compartmentalized and eventually leads to discrete tooth fields. Thus, both whole mount data and 3D reconstructions show the separation of initially compact tooth-competent zones into distinct tooth fields. Therefore, the two dental arcades of the axolotl dentition clearly originate from a single tooth-competent zone.

### Further Differences Among Axolotl Dental Arcades Arise by a Field-Specific Addition of Tooth Germs

**FIGURE 3:**
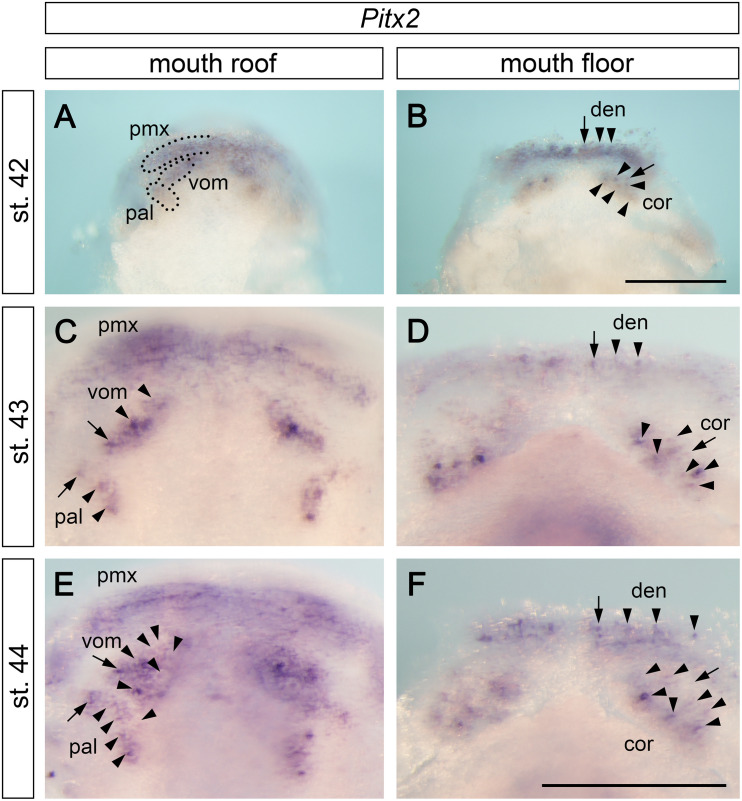
Expression pattern of *Pitx2* at later stages of axolotl odontogenesis. *Pitx2* expression becomes gradually restricted to individual tooth germs (arrows mark initiator-tooth germs of respective tooth fields and arrowheads mark sequentially added tooth germs). **(A,C,E)** Within the mouth roof epithelium, the initial *Pitx2* expression delineating individual tooth fields (black broken lines in **A**) becomes focused into individual tooth germs, inter-germ regions, and the forming successional dental laminae of vomerine (vom) and palatine (pal) tooth fields **(C,E)**. *Pitx2* expression is further associated with the developing premaxillary tooth field (pmx), although individual tooth germs cannot be discerned. **(B,D,F)** Within the mouth floor epithelium, strong *Pitx2* expression is visible in the developing tooth germs of dentary (den) and coronoid (cor) tooth fields. cor, coronoid tooth field; den, dentary tooth field; pal, palatine tooth field; pmx, premaxillary tooth field; vom, vomerine tooth field. Scale bars equal 500 μm (scale bar in **B** is valid for **A,B**, scale bar in **F** is valid for **C–F**).

The separation of tooth-competent zones into individual tooth fields is followed by a progressive and rapid addition of tooth germs within individual fields. *Pitx2* expression progressively changes from broadly delineating the tooth-competent zones into focal patterns that label individual tooth germs (compare the gradual change in *Pitx2* expression portrayed on Figures 2M,O, 3A–D). Interestingly, this change in *Pitx2* expression takes place earlier on the mouth floor than on the mouth roof; i.e., stage 42 shows that *Pitx2* is expressed generally within premaxillary, vomerine, and palatine fields (Figure 3A) and focally within tooth germs of the dentary and coronoid fields (Figure 3B). This suggests that the development of tooth fields on the mouth floor is slightly advanced relative to that on the mouth roof.

**FIGURE 4:**
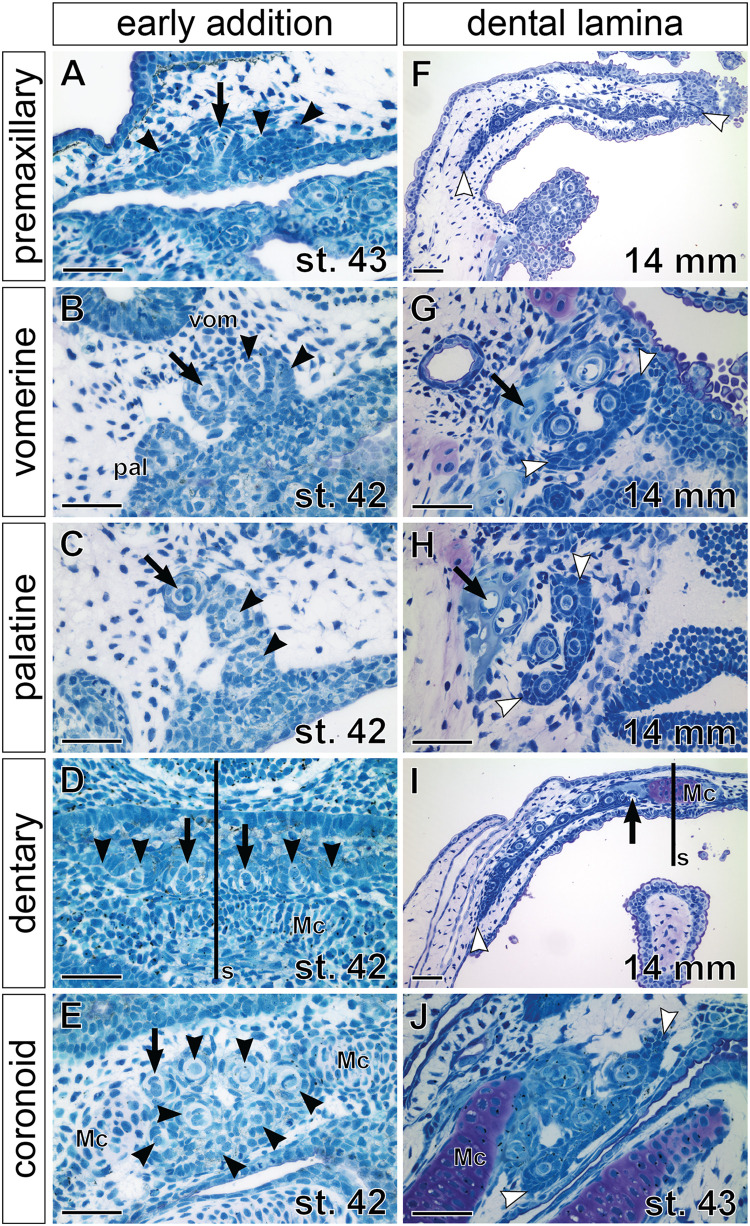
Histological analysis of tooth addition within axolotl tooth fields. Horizontal sections (left sides shown, anterior to the top) through individual tooth fields show positions of the respective initiator-tooth (black arrows) and the newly added successive tooth germs (black arrowheads) at stages of early addition **(A–E)** and later morphogenesis **(F–J)**, when successional dental laminae are clearly discernible (demarcated by white arrowheads). **(A)** Premaxillary tooth field at stage of opened mouth (stage 43) contains three tooth germs (arrowheads) that are added laterally and medially to the initiator-tooth (arrow). **(B,C)** Vomerine (vom) and palatine (pal) tooth fields at stage 42 are composed of laterally positioned initiator-teeth (arrow) and two adjacent medially positioned tooth germs (arrowheads). **(D)** Dentary tooth fields are each initiated from the initiator-tooth (arrows) developing close to the mandibular symphysis (s) and new tooth germs (arrowheads) are added laterally along the Meckel’s cartilage (Mc). **(E)** Coronoid tooth field is composed of a patch of tooth germs (arrowheads) with the initiator-tooth present antero-laterally (arrow). **(F)** Premaxillary tooth field of a 14 mm TL larva shows arrangement into a tooth row. New tooth germs are added lingually from the successional dental lamina and, as the upper jaw grows, the premaxillary field expands by further addition of tooth germs at lateral and medial edges of the dental lamina (white arrowheads). **(G,H)** Vomerine and palatine tooth fields containing several teeth arranged into a tooth patch with new germs arising from the lingually positioned successional dental laminae (white arrowheads). Slight difference in composition exists between these tooth fields that can be ascribed due to the addition of first teeth antero-medially to the initiator-tooth (black arrow) in the vomerine field **(B)** and postero-medially in the palatine field **(C)**, and to the subsequent development of new tooth germs from the lingually placed successional dental laminae (white arrowheads). **(I)** Dentary tooth field is arranged into the tooth row. New tooth germs are added from the lingually positioned successional dental lamina and laterally (white arrowhead) due to expansion of the growing Meckel’s cartilage (Mc). **(J)** Coronoid tooth field at a slightly older stage than that of **(E)** develops from the lingual successional dental lamina (white arrowheads). Scale bars equal 100 μm.

Stages around hatching display a continuation of the addition of new tooth germs within individual tooth fields but also differences among their prospective single-rowed versus multi-rowed arrangements. Moreover, while new germs initially arise from the adjacent superficial epithelium (Figures 4A–E), later tooth germs develop from the invaginated successional dental laminae, whose positions regulate the future arrangement of the respective tooth fields (Figures 4F–J). For example, in the premaxillary field, new tooth germs are added laterally and medially to the initiator-teeth (Figure 4A), while in the dentary field, new germs are added laterally from the pair of parasymphyseal initiator-teeth (Figures 2L,P, 4D). Though formed differently, both fields eventually produce teeth assembled into a single row that runs along the jaw margins and is connected lingually through a continuous dental lamina (Figures 4F,I). Likewise, the tooth fields on the mouth roof produce initial rows of tooth germs by antero-medial addition in vomerine fields and postero-medial addition in palatine fields (cf. Figures 3C, 4B,C). The lingual positions of successional dental laminae then result in the addition of new germs lingually (Figures 3E, 4G,H), thus producing multi-rowed patches of teeth on the mouth roof. On the mouth floor, the coronoid tooth field is produced first by the sequential addition of tooth germs from the adjacent epithelium followed by addition from the lingually positioned successional dental lamina (Figures 4E,J). Thus, the pattern of the addition of tooth germs sets a basis for the arrangement of axolotl dentition into outer and inner dental arcades with different properties. During larval development, teeth of the outer dental arcade become arranged into a single functional row while teeth of the inner dental arcade become clustered into patches with many functional rows.

### Axolotl Dentition Arises at the Ecto-Endodermal Boundary

**FIGURE 5:**
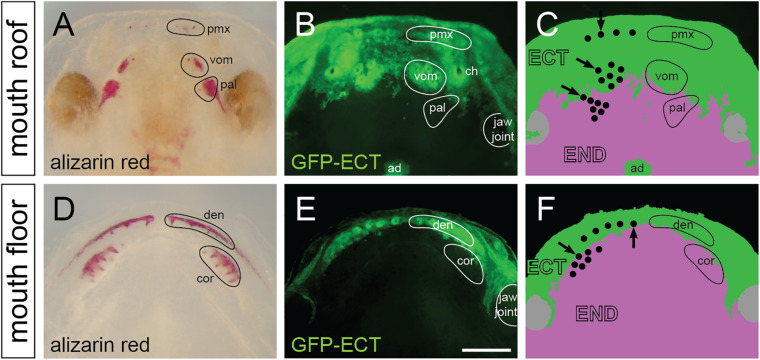
Distribution and epithelial derivation of teeth in the axolotl oral cavity. **(A,D)** Whole mount larvae at stage 44 dissected at the jaw joint and stained by alizarin red demonstrate developing teeth assembled into premaxillary (pmx), vomerine (vom), and palatine (pal) tooth fields on the mouth roof **(A)**, and into dentary (den) and coronoid (cor) tooth fields on the mouth floor **(D)**. **(B,C,E,F)** Epithelial origin of teeth and schematic interpretation of germ-layer distribution in the axolotl oral cavity (note that only the basal epithelial layer is depicted; the apical layer of the oral epithelium is derived from endoderm). Black dots in **C** and **F** represent individual teeth, and positions of initiator-teeth of each tooth field are labeled with black arrows. Teeth developing at the ecto-endoderm boundary are found in vomerine, palatine, dentary, and coronoid tooth fields. ad, adenohypophysis; ch, choana (inner nostril); den, dentary teeth; ECT, ectoderm; END, endoderm; pal, palatine teeth; pmx, premaxillary teeth; cor, coronoid teeth; vom, vomerine teeth. Scale bar equals 500 μm.

The distribution of ectoderm and endoderm in the oropharynx has been hypothesized to play a key role in the initiation and patterning of teeth (Smith, 2003; Ohazama et al., 2010), yet, a thorough knowledge on the distribution of these epithelia in extant vertebrates is largely unknown (reviewed in Soukup et al., 2013). We previously studied the distribution of these epithelia in the Mexican axolotl and showed that the labial portion of the nascent mouth epithelium consists of the ectodermal basal layer and the endodermal apical layer and that the lingual ectodermal boundary runs through the tooth fields (Soukup et al., 2008). We performed a new series of transplantations of the prospective oral ectoderm from GFP-transgenic axolotl neurulae into white hosts (*n* = 8). Experiments resulted in an invariable labeling of the ectodermal lining inside the oral cavity, which allows a detailed visualization of the ecto-endodermal boundary during the developing dentition. On whole mounts, the GFP-labeled ectoderm reaches an anterior portion of the palate in front of and slightly behind inner nostrils (Figure 5A) and the labial portion of the lower jaw (Figure 5D). The ecto-endodermal boundary on the palate stereotypically displays an uneven, roughly bilaterally symmetrical shape with an intermingling of cells, especially medially at the place of migration of the adenohypophyseal anlage (Figures 5B,C). On the other hand, the ecto-endodermal boundary on the mouth floor displays a crescent-shaped arrangement that runs along the length of the lower jaw (Figures 5E,F).

**FIGURE 6:**
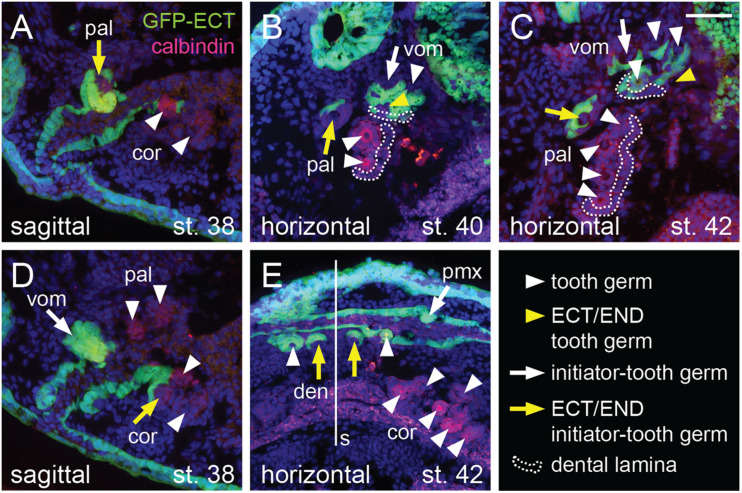
Histological analysis of the epithelial origin of the axolotl teeth. Histological analysis shows details of epithelial origin and modes of addition of teeth (marked by anti-calbindin antibody) within the tooth fields. Arrows mark initiator-tooth germs in each tooth field, arrowheads mark sequentially added tooth germs, yellow arrow/arrowhead denotes tooth developing at the ecto-endoderm boundary, and white arrow/arrowhead denotes tooth developing solely from either ectodermal or endodermal epithelium. **(A)** Sagittal section showing lateral-most palatine tooth developing at the ecto-endoderm boundary (yellow arrow) close to endoderm-derived coronoid tooth germs (white arrowheads). **(B)** Horizontal section through vomerine and palatine tooth fields. Two vomerine teeth develop in the ectodermal epithelium (white arrow and arrowhead) and one tooth germ is of ecto-endodermal origin (yellow arrowhead). Palatine tooth field is composed of one ecto-endodermal tooth germ labially (yellow arrow) and two endodermal germs (white arrowheads) lingually. Note the just-arising successional dental laminae (broken lines) that are of endodermal origin in case of the palatine field and ecto-endodermal origin in case of the vomerine field. **(C)** Horizontal section of later stage vomerine and palatine tooth fields showing ecto-endodermal germ in each vomerine and palatine fields (yellow arrow and arrowhead) next to other ectodermal vomerine teeth and endodermal palatine teeth (white arrowheads). Successional dental laminae (broken lines) are of ecto-endodermal origin in vomerine field and endodermal origin in palatine field. **(D)** Sagittal section medial to that shown in **(A)** depicting ecto-endodermal coronoid tooth (yellow arrow), which develops at the level of other tooth germs of vomerine, palatine, and coronoid tooth fields (white arrow and arrowheads). **(E)** Horizontal section showing ecto-endodermal teeth developing medially in the dentary tooth field (yellow arrows) next to other ectodermal teeth of dentary and premaxillary fields and endodermal teeth of the coronoid field (white arrowheads). den, dentary teeth; pal, palatine teeth; pmx, premaxillary teeth; cor, coronoid teeth; s, symphysis; vom, vomerine teeth. Scale bar equals 50 μm.

The ecto-endodermal boundary runs through the tooth fields so that individual tooth germs display a differential epithelial origin: ectodermal, endodermal, or ecto-endodermal. Histological analysis shows that the dentary tooth fields are composed of ecto-endodermal germs developing close to the mandibular symphysis (Figure 6E, yellow arrows). The other dentary teeth, on the other hand, develop from the ectodermal epithelium laterally along the jaw (Figure 6E, white arrowheads). Interestingly, the parasymphyseal teeth represent the initiator-teeth from where new germs are added laterally, i.e., the dentary field is initiated directly at the ecto-endodermal boundary. Single ecto-endodermal tooth germs are also found antero-laterally in the coronoid and palatine tooth fields, while other teeth of these fields are of endodermal origin (Figures 6A–D, yellow arrows). Based on histological data and *Shh* expression (Figures 2F,J, 3E, 4C,H), we reason that the ecto-endodermal teeth represent the initiator-teeth of these fields and that other teeth are added lingually from endodermal regions. Similarly to the dentary field, the initiator-teeth of the coronoid and palatine fields are found at the ecto-endodermal boundary. The initiator-tooth and also the first added tooth germs of the vomerine field, on the other hand, develop anterior to the ecto-endodermal boundary, i.e., from the ectodermal epithelium (Figure 6B, white arrow and white arrowheads). Yet, some teeth arising at later larval stages develop from ecto-endoderm due to the lingually positioned ecto-endodermal successional dental lamina (Figures 6B,C, yellow arrowheads). In contrast to other tooth fields, the premaxillary field is composed solely of teeth of ectodermal origin (Figures 5B,C).

**FIGURE 7:**
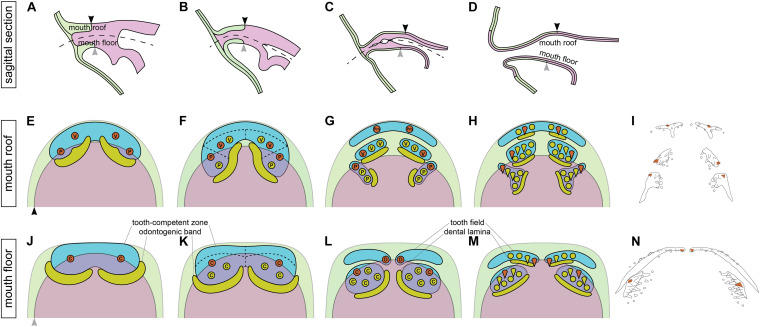
Relations between the germ-layer distribution and patterning of the axolotl dentition. During the course of the axolotl mouth development, oral ectoderm and endoderm do not form a sharp boundary, but instead the basal layer of oral ectoderm involutes to constitute the basal layer of the nascent oral epithelium while the solid endoderm forms the apical layer **(A–D)**. As a result, the ecto-endodermal boundary within the basal layer of the mouth roof (black arrowhead) and floor (gray arrowhead) is different from that within the apical layer. The broken line in **(A–C)** shows the place of the future mouth cavity. Tooth-competent zones become established in the basal epithelial layer of the mouth roof and floor (blue regions in **E,F,J,K**) and later become separated into the prospective tooth fields (blue regions in **G,H,L,M**). Tooth germs arise progressively during the process of this separation. Orange circles mark the positions of the initiator-teeth of each prospective tooth field and yellow circles denote positions of successively added tooth germs. Yellow regions mark positions of odontogenic bands at earlier stages **(E,F,J,K)** and successional dental laminae at later stages **(G,H,L,M)**. Green and magenta regions show the extent of oral ectoderm and endoderm, respectively. Only the basal epithelial layer, i.e., the tooth-forming layer of the double-layered epithelium, is depicted (the black and gray arrowheads in **E,J** correspond to those in **A–D**). First, tooth germs of vomerine (V), palatine (P), and coronoid (C) tooth fields appear prior to those of the premaxillary (Pm) and dentary (D) tooth fields. The initiator-teeth of palatine, coronoid, and dentary tooth fields stereotypically arise at the boundary of ectoderm (green) and endoderm (magenta) (orange circles in **F,K,L**), while the initiator-teeth of the premaxillary and vomerine tooth fields arise from ectoderm (orange circles in **E,G**). The successive tooth germs arise from ectoderm in the case of premaxillary and dentary tooth fields, from endoderm in the case of palatine and coronoid tooth fields, and from ectoderm or ecto-endoderm in the case of vomerine tooth fields **(F–H,K–M)**. These early events in oro- and odontogenesis lead to the salamander-specific distribution of teeth on the bones of the upper and lower jaws and on the palate **(I,N)**. Orange teeth in **(I,N)** represent the initiator-teeth of each tooth field. **(I,N)** are schematized Figures 1B,C, respectively. den, dentary; pal, palatine; pmx, premaxillary; cor, coronoid; vom, vomerine.

The combination of data on the modes of the addition of new tooth germs within the tooth fields and their germ-layer derivation thus allows us to propose that the initiator-teeth in three tooth fields arise directly at the boundary between ectoderm and endoderm (Figures 7A,E,G) and those of the other two are initiated in ectodermal regions. No initiator-tooth stereotypically develops from the endoderm. New tooth germs subsequently arise from ectoderm, endoderm or ecto-endoderm due to specific development of the respective tooth field.

## Discussion

Based on the presented data, we evaluate the topic on how vertebrate dentitions are established during development. We show that the typical tetrapod dentition composed of many tooth fields and assembled into dental arcades, such as that present in the axolotl, originates from an initially compact *Pitx2*-positive tooth-competent zone on both the mouth roof and the floor. The tooth-competent zone becomes progressively compartmentalized until, by the time of hatching, the larva possesses several well-defined tooth fields. Field-specific differences in tooth addition and patterning lead to the single-rowed versus multi-rowed tooth arrangements. In combination with fate mapping, we further show that the initial tooth-competent zone colocalizes with the boundary between ectoderm and endoderm and that this interaction instigates the initiation and early events of odontogenesis.

### Axolotl Tooth Fields Arise by Separation of Tooth-Competent Zone

The initial state of axolotl dentition is evidenced by *Pitx2*-expressing tooth-competent zones that are overlain by *Shh*-expressing odontogenic bands at their posterior portions (Figures 7A,E). This initial co-expression of *Pitx2* and *Shh* corresponds well to the situation found on the mouth roof and floor of other vertebrates such as shark, trout, cichlids, tetra, vole, and mouse, and it probably represents a blueprint of the developing oral dentitions in vertebrates (Keränen et al., 1999; Fraser et al., 2004, 2008; Stock et al., 2006; Debiais-Thibaud et al., 2015; Rasch et al., 2016). Concordantly, the lack of co-expression of these two factors has been ascribed to the inability to form oral teeth in zebrafish or to initiate a third tooth row of the two-rowed dentition in the cichlid *Cynotilapia afra* (Stock et al., 2006; Fraser et al., 2008). In cichlids, the following reiterated focal expression of *Shh* within the solid *Pitx2*-expressing domain defines positions of newly added tooth germs that lead to the development of dentitions composed of many teeth (Fraser et al., 2008). Likewise, new foci of *Shh* expression within the *Pitx2*-expressing tooth-competent zones demarcate the newly added tooth germs also in axolotl (Figures 7A,B,E,F). However, both the positioning of new tooth germs and the odontogenic bands occurs even prior to the complete separation of tooth-competent zones.

The progressive compartmentalization of tooth-competent zones leads to individual tooth fields whereas no tooth field is established *de novo*. This means that in axolotl, the dentition composed of five pairs of tooth fields and assembled into outer and inner dental arcades develops only from two *Pitx2*-expressing tooth-competent zones present on the mouth roof and floor. The progressive compartmentalization is reminiscent of the proposed sequential partitioning of tooth-competent zones during the establishment of the heterodont dentition in mammals, where a gene toolbox reiteratively functions in a nested fashion to progressively partition the previously defined regions into smaller compartments up to individual tooth cusps (Jernvall and Thesleff, 2000). Under this scenario, a field of competence (odontogenic band) is first established; second, a tooth type area is specified (incisor, canine, premolar, and molar); third, individual teeth are initiated; and finally, their cusps arise. By applying this scenario to homodont dentitions composed of many uniform teeth, such as those found in the axolotl or, e.g., ray-finned fishes, the initially defined tooth-competent region would directly be compartmentalized into individual tooth germs (Streelman et al., 2003; Fraser et al., 2008). In axolotl, however, the tooth-competent zones give rise not only to the marginal teeth, for which the model of progressive partitioning was originally proposed, but also to the entire tetrapod-type dentition composed of many tooth fields assembled into two dental arcades.

Interestingly, partitioning of the axolotl tooth-competent zones into prospective tooth fields also affects the odontogenic bands. The initiator-tooth germs of vomerine and palatine fields are among the first appearing teeth of the axolotl dentition, and these fields initially share a common odontogenic band (Figures 7A,B). However, during the process of partitioning of tooth-competent zones and the appearance of distinct vomerine and palatine tooth fields, the single odontogenic band becomes divided into vomerine and palatine parts. Both parts produce replacement teeth of their respective field. In this respect, it is interesting to note that the early intimate relation between the vomerine and palatine odontogenic bands may explain the later association of vomerine and palatine successional dental laminae in larval salamanders. Although this is not the case in the axolotl, where the vomerine and palatine tooth fields develop separately (see Clemen and Greven, 1977), the mature tooth-producing vomerine and palatine successional dental laminae of the fire salamander are joined by an unproductive epithelial band (Clemen, 1978). Thus, in the fire salamander, the connection between the vomerine and palatine tooth fields may be a continuation from early developmental stages, where these two tooth fields share a common odontogenic band.

### Axolotl Inner Dental Arcade Is Initiated Prior to the Outer Dental Arcade

Tooth fields in axolotl emerge in the following order: (1) vomerine + palatine + coronoid, (2) dentary, (3) premaxillary, i.e., teeth of the inner dental arcade are initiated prior to those of the outer arcade (Figure 2). The initial sequence of appearance is reflected in the composition of the dentition at hatching (stage 44), when the tooth fields of the inner arcade show a multi-rowed arrangement, while those of the outer arcade still undergo addition of new germs into a row. This developmental progression of the inner over the outer dental arcade is conspicuous. In general, during embryonic and larval periods, the differentiation of body parts such as the pharyngeal region, rhombomeres, or somites, usually progresses in an anterior-to-posterior fashion (Dequéant and Pourquié, 2008; Graham, 2008; Schilling, 2008). Deviations of this rule have been associated with the development of adaptive organs necessary for embryonic or larval survival (Pospisilova et al., 2019; Stundl et al., 2019, 2020). Salamander larvae, including the neotenic Mexican axolotl, are suction feeders. After being sucked in, the prey is firmly gripped in the oral cavity and then oriented through several cycles of releasing, reorienting, and immediate re-gripping until swallowing (Deban and Wake, 2000). This manipulation is facilitated by the presence of tooth patches at the rear of the mouth. Concordantly, the first odontogenic bands and successional dental laminae, from which subsequent tooth germs arise in axolotl, develop in relation to vomerine, palatine, and coronoid tooth fields, thus facilitating the early achievement of a multi-rowed arrangement of the inner dental arcade. The early establishment of tooth patches in the inner dental arcade assures that the newly hatched axolotl larva is equipped with an apparatus ready-to-use for capturing and processing prey, whereas the outer arcade may be lagging in time and develop fully only at later larval stages.

### The Field-Specific Addition of Teeth Controls the Final Composition of the Axolotl Dentition

The pattern of tooth germ addition is disparate among individual axolotl tooth fields (Figure 7). Generally, the final appearance of the respective tooth field depends on the shape of the *Pitx2*-expressing field and the position of the initiator-tooth. For example, the dentary field is narrow and strand-shaped, wherein tooth germs are sequentially added into a row, and this situation is reminiscent of the developing mammalian dentition. The coronoid field, on the other hand, is wide and tooth germs assemble into a cluster (Figures 7G,H), i.e., a situation reminiscent of the multi-rowed dentition of cichlids (Fraser et al., 2008). The differences in tooth patterning at early stages of axolotl development are subsequently reflected in the final arrangement of teeth at larval stages.

Each prospective tooth field in the axolotl starts its development with the appearance of the first tooth. In zebrafish, the first tooth acts as a signaling center for the initiation of two adjacent tooth germs, and these three teeth then constitute the first row of the dentition (Van der heyden and Huysseune, 2000; Gibert et al., 2019). In the mouse, the first tooth germ similarly initiates the whole molar row (Prochazka et al., 2010; Sadier et al., 2019). The first tooth thus acts as an initiator cue for the development of the whole tooth row or tooth patch (Sadier et al., 2020) while spacing between the teeth is mediated by the zone of inhibition surrounding each tooth germ (Osborn, 1978; Huysseune and Witten, 2006; Fraser et al., 2008). In the axolotl, the position of the initiator-tooth is specific to each tooth field (Figure 7) and these different positions next trigger disparate ways, in which new germs are added (see Huysseune and Witten, 2006). For example, among the narrow strand-shaped fields, the dentary tooth germs are added laterally to the parasymphyseally positioned initiator-tooth while the premaxillary tooth germs are added both laterally and medially to the mid-laterally positioned initiator-tooth (Figures 7C,D,G,H). The addition of new germs within each tooth field progresses through redeployment of tooth-competent genes, such as *Shh*, *Bmp2*, or *Bmp7*, until this region becomes fully filled up with tooth germs (Figures 2, 3 and Supplementary Figure 2).

After filling up the initial area of the tooth field with individual teeth, new tooth germs can only be added by further enlargement of this field. This may happen basically in two ways that can act simultaneously. First, the embryonic growth leads to the enlargement of the field by a medio-lateral prolongation, and so new tooth germs may be added sequentially along the jaw length. This is most notably visible on the dentary, but also premaxillary and maxillary, where the lateral addition of new teeth of the premaxillary field progressively covers the maxillary bone, resulting in the presence of the common dental lamina on the upper jaw margin (Clemen and Greven, 1977, 1994). Second, the lingually positioned successional dental lamina provides further space for the additional production of tooth germs behind the already formed tooth row(s). This is most conspicuous in case of vomerine or palatine tooth fields. In vertebrates, progressive enlargements of fields of competence have been associated with patterning of other, non-dental, iterative structures such as bird plumage, or shark denticles (Jung et al., 1998; Cooper et al., 2018) and the addition of new units into the existing row simultaneously with the addition of new rows thus seems to be a general phenomenon of dentitions composed of many teeth and tooth generations (Van der heyden and Huysseune, 2000; Ellis et al., 2016). From this viewpoint, the addition of new tooth units simultaneously at the edges of the tooth fields and from successional dental laminae in the axolotl is in good accord with that present in other vertebrates.

### Some Axolotl Teeth Develop Stereotypically at the Ecto-Endodermal Boundary

The boundary between ectoderm and endoderm in axolotl runs through individual tooth fields (Figure 7). Except for the purely ectoderm-derived premaxillary tooth field that later expands laterally onto the maxillary bones (Clemen and Greven, 1977), the remaining tooth fields are ecto-endodermal. In palatine, coronoid, and dentary tooth fields, the relation between the ecto-endodermal boundary and the teeth is special in that the boundary stereotypically runs through the first-forming tooth germs, i.e., initiator-teeth of these fields. Thus, in axolotl, the initiator-tooth either develops from the ectoderm (premaxillary and vomerine) or directly at the ecto-endodermal boundary (palatine, coronoid, dentary, Figure 7, orange circles). We found no purely endoderm-derived initiator-tooth. Interestingly, among the fields with the ecto-endodermal initiator tooth, the newly added tooth germs develop either from ectoderm (dentary field) or from endoderm (palatine and coronoid fields). In the vomerine field, where the initiator-tooth is ectodermal, the subsequent germs are ectodermal or ecto-endodermal.

The ecto-endodermal boundary runs through the tooth fields in such a way that even the odontogenic bands of early larvae and the successional dental laminae that produce new tooth germs at later larval stages may be of double-germ layer origin. Besides the ectodermal dental lamina of the premaxillary field and the endodermal dental laminae of the palatine and coronoid fields, the dental lamina of the vomerine field is ecto-endodermal (Figures 6B,C), and we propose its presence also in the case of the dentary field.

With spatial relations between the germ layer distribution and the initiator-tooth development, the axolotl constitutes a unique model among extant vertebrates. In the mouse, the *Sox17*-reporter line shows the presence of the *Sox17*-positive endodermal epithelium at the rear of the tongue and posterior to the developing dentition, meaning that all murine teeth are ectoderm-derived (Rothova et al., 2012). In zebrafish, the tooth-forming pharyngeal epithelium *per se* is endodermal, although teeth develop only when the pharyngeal endoderm contacts the surface ectoderm via the pharyngeal cleft/pouch and only after an apical periderm-like layer on top of the odontogenic epithelium was formed (Oralová et al., 2020). In the axolotl, mouth development progresses through a stomodeal collar, where the oral ectoderm involutes and eventually contributes, in the anterior oral regions, exclusively to the basal layer of the double-layered oral epithelium (Figures 7A–D; Soukup et al., 2008, 2013). Given that initiator-teeth of individual axolotl tooth fields are either fully ectodermal or half ectodermal and half endodermal, this suggests that the ectoderm may be responsible for the initiation of axolotl dentition. Why should, instead of staying externally, the oral ectoderm progress through a complicated stomodeal collar morphogenesis, rather than reach the prospective palatal and coronoid positions to initiate teeth? Such an interpretation would, together with the data on the role of germ layers during the initiation of teeth in zebrafish (Oralová et al., 2020), be mechanistically well in line with the modified outside-in hypothesis of tooth origin. This hypothesis suggests that the odontogenic potential originated externally in the skin, where the surface ectoderm instigated development of odontodes. This odontogenic potential later became translocated into the oropharyngeal region in a way that teeth of extant vertebrates develop either from the ectoderm or from the endoderm, yet with a close-by presence of the tooth-instructive ectoderm (Huysseune et al., 2009, 2010). Conversely, *in vitro* experiments, in which different embryonic tissues from salamanders were combined and their differentiation potential was studied, corroborate that tooth germs are produced only when oral ectoderm, foregut endoderm, and cranial neural crest are co-cultured (Wilde, 1955; Graveson et al., 1997). No teeth form when either oral ectoderm or foregut endoderm alone is co-cultured with neural crest (Wilde, 1955; Takata, 1960). The combinatorial presence of ectoderm, endoderm, and neural crest thus seems to be a necessary prerequisite for salamander tooth development.

Recently, an evolutionary–developmental relationship between teeth and taste buds has been hypothesized based on shared expression profiles (Fraser et al., 2010). The relation between these two structures has further been linked due to the shared niche of progenitor cells present at the taste–tooth junction in the shark, ray, pufferfish, and leopard gecko (Martin et al., 2016; Thiery et al., 2017; Salomies et al., 2019; Rasch et al., 2020), suggesting that this relationship could be present also in the axolotl. Taste buds in axolotl arise from the basal layer of the oropharyngeal epithelium, and thus can be both ectodermal and endodermal (Barlow and Northcutt, 1995; Barlow, 2000). Interestingly, although the relationship between teeth and taste buds has not directly been studied in the axolotl, the density of taste buds within the oropharyngeal cavity is highest close to the tooth fields (Northcutt et al., 2000). If the presence of the taste–tooth junction were confirmed in the axolotl, it would further be interesting to assess its relations to the ecto-endodermal boundary. In such a case, we would predict colocalization of the taste–tooth junctions and the ecto-endodermal boundary close to the dentary and vomerine tooth fields, i.e., places where this boundary resides lingual to the respective tooth field (Figures 5B,C,E,F), a hypothesis ready to be tested. Furthermore, ecto-endodermal fate mapping performed on species, where the taste–tooth junction was proposed, would further test the plausibility of this hypothesis.

Our data lead us to reason that the boundary between the ectoderm and endoderm accounts for a potential source of instruction factors that stimulate the onset of the odontogenic program. Such an interaction system with a potential tooth-instructing role may be a widespread feature of the vertebrate oropharyngeal cavities especially at places, where the outer ectoderm comes into contact with the inner endoderm. For example, if the physical ecto-endodermal contact is compromised *in vivo* in zebrafish, the odontogenic program is not triggered (Oralová et al., 2020). Based on these data, we hypothesize that the combinatorial influence of the ectoderm and endoderm on tooth development, or the physical boundary of these epithelia *per se*, may be a much more widespread and perhaps ancient feature of vertebrate odontogenesis. Is there a support for an ancient role of the ecto-endodermal boundary in tooth initiation? Interestingly, the basalmost stem osteichthyan *Lophosteus* shows initiation of dermal odontodes and teeth at the oral–dermal boundary running along the length of the lower jaw (Chen et al., 2020). New odontodes are sequentially added labially and new teeth lingually from this boundary. Presumably, this boundary represents a generative interface of the outer and inner developmental environments from where new hard tissue elements (odontodes/teeth) develop in an ordered way (Chen et al., 2020). Although the fossil data cannot provide the precise knowledge on the distribution of soft tissues such as tooth-forming epithelia and the direct linkage of the oral-dermal and ecto-endodermal boundaries, it is tempting to speculate that the oral-dermal boundary on the lower jaw of *Lophosteus* represents an ancient ecto-endodermal boundary with instructive role for the initiation of tooth development. In this context and in the context of the current study, there is an apparent need for the broader use of detailed fate-mapping studies in the range of “models” and “non-models” in order to visualize and evaluate the role of the ecto-endoderm boundary for the initiation of vertebrate odontogenesis.

## Conclusion

Vertebrates display a wide array of dentitions with patterned arrangements. Yet, how such patterns emerge during development and what the initiation agents of tooth patterning are, are largely unanswered questions (Smith, 2003; Huysseune and Witten, 2006; Fraser et al., 2008; Balic, 2019). The axolotl represents a suitable model for the study of patterning of teeth into a row or a patch. Moreover, in this animal, the colocalization of the initiator-teeth and the ecto-endodermal boundary points to the boundary as the potential source of initiation signals that instigate odontogenesis. Be it directly at the boundary or in its proximity, vertebrate dentitions frequently develop at places of interactions between ectoderm and endoderm, such as the jaws, the palate, or the branchial arches. Therefore, the role of the ecto-endodermal boundary on tooth initiation and development is certainly a challenging yet vastly underestimated topic of vertebrate embryogenesis.

## Data Availability Statement

The datasets presented in this study can be found in online repositories. The names of the repository/repositories and accession number(s) can be found in the article/Supplementary Material.

## Ethics Statement

Ethical review and approval was not required for the animal study because the presented experimental work was performed on pre-hatching embryos. No post-hatching experiment has been done. No transgenic lines were generated.

## Author Contributions

VS and RC conceived, designed the experiments, and analyzed the data. VS, AT, YY, AP, and RC performed the experiments and generated the data. AT, H-HE, EMT, and RC provided technological support and provided advice on molecular biology and *in situ* hybridization. RC administered the project. VS wrote the original draft of the manuscript. VS, H-HE, and RC reviewed and edited the final version of the manuscript. All authors read and approved the final version of the manuscript.

## Conflict of Interest

The authors declare that the research was conducted in the absence of any commercial or financial relationships that could be construed as a potential conflict of interest.

## Supplementary Material

Supplementary Figure 1 Expression of selected genes during axolotl odontogenesis. **(A–D)** Expression of *Bmp2* and *Bmp7* demarks positions of individual initiator-tooth germs of each nascent tooth field (arrows) and positions of successive tooth germs (arrowheads) in a pattern similar to that of *Shh* (see Figure 2). **(E,F)** Expression of *Dlx3*, on the other hand, delineates positions of tooth fields (black broken lines) similarly to that of *Pitx2* (see Figure 2). **(G,H)** *Dlx5* transcripts are restricted to the developing tooth germs besides their presence at other craniofacial regions. cor, coronoid field; den, dentary field; pal, palatine field; pmx, premaxillary field; vom, vomerine field. Scale bar equals 500 μm.

Supplementary Figure 2 3D models of the developing axolotl dentition based on serial sagittal sections hybridized with the *Pitx2* probe. The models visualize the shape and relationship of *Pitx2*-expressing areas aka tooth competent zones and tooth fields. During embryonic development, the initially compact tooth-competent zones on the mouth roof and floor (st. 38) become compartmentalized into individual tooth fields. The models were reconstructed based on the complete series of sagittal sections hybridized with a *Pitx2* probe (left column, solid lines mark the basement membrane of the oropharyngeal epithelia, broken lines mark the place of future oropharyngeal opening). Mouth roof expression is in red color, mouth floor expression in blue color, basal epithelial layer of the oral epithelium in gray color and cyan dots demark positions of tooth germs. ad, adenohypophysis; cor, coronoid field; den, dentary field; n, nasal cavity; pal, palatine field; pmx, premaxillary field; vom, vomerine field. Scale bar equals 100 μm.
