# Supplementary figures and images for: Oral and Palatal Dentition of Axolotl Arises From a Common Tooth-Competent Zone Along the Ecto-Endodermal Boundary
Source: Front Cell Dev Biol. 2021 Jan 11;8:622308. doi: 10.3389/fcell.2020.622308 (PMC7829593; doi:10.3389/fcell.2020.622308)

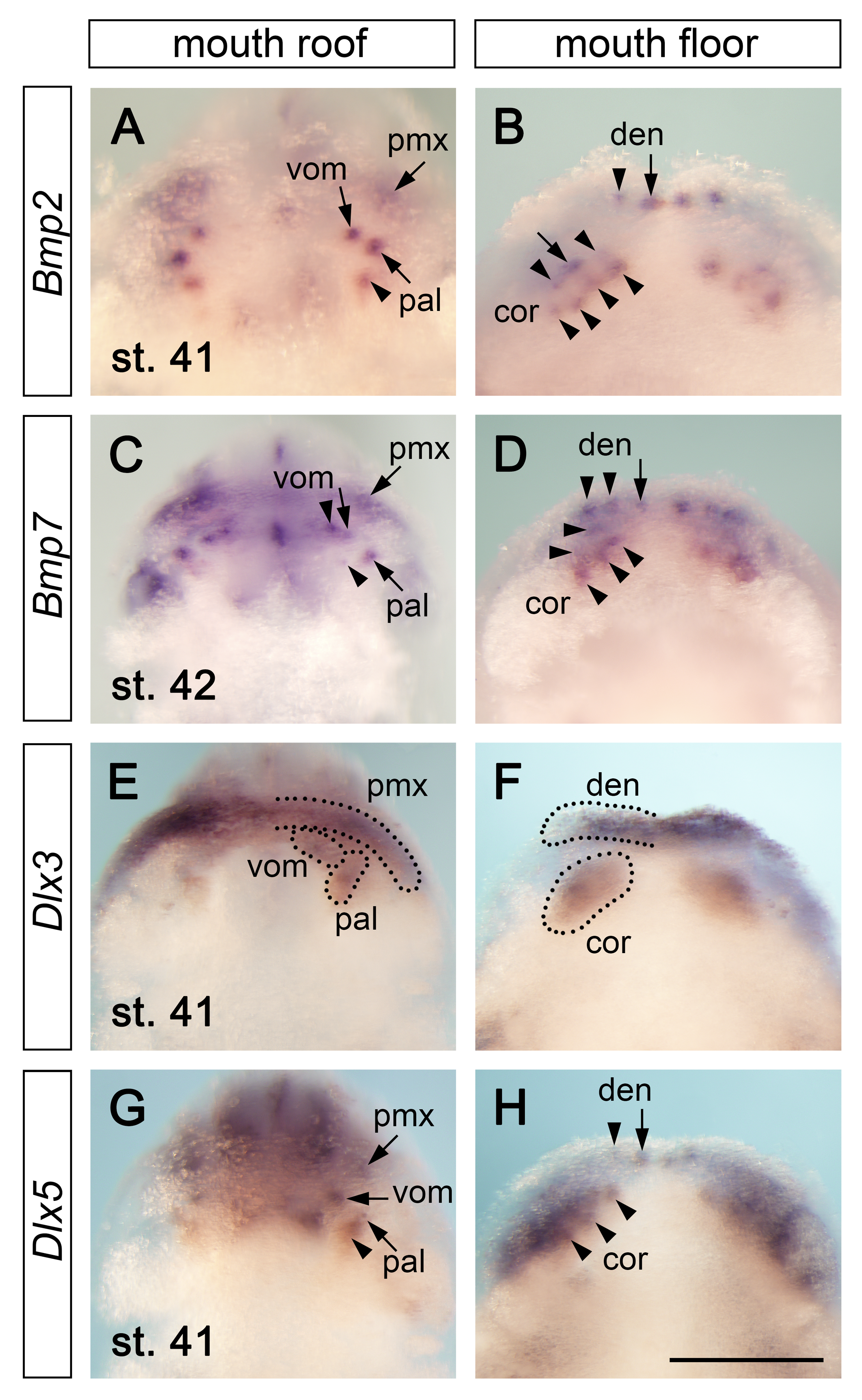

Supplement: Supplementary Figure 1 — Expression of selected genes during axolotl odontogenesis. (A–D) Expression of Bmp2 and Bmp7 demarks positions of individual initiator-tooth germs of each nascent tooth field (arrows) and positions of successive tooth germs (arrowheads) in a pattern similar to that of Shh (see Figure 2). (E,F) Expression of Dlx3, on the other hand, delineates positions of tooth fields (black broken lines) similarly to that of Pitx2 (see Figure 2). (G,H) Dlx5 transcripts are restricted to the developing tooth germs besides their presence at other craniofacial regions. cor, coronoid field; den, dentary field; pal, palatine field; pmx, premaxillary field; vom, vomerine field. Scale bar equals 500 μm. [file Image_1.TIF]

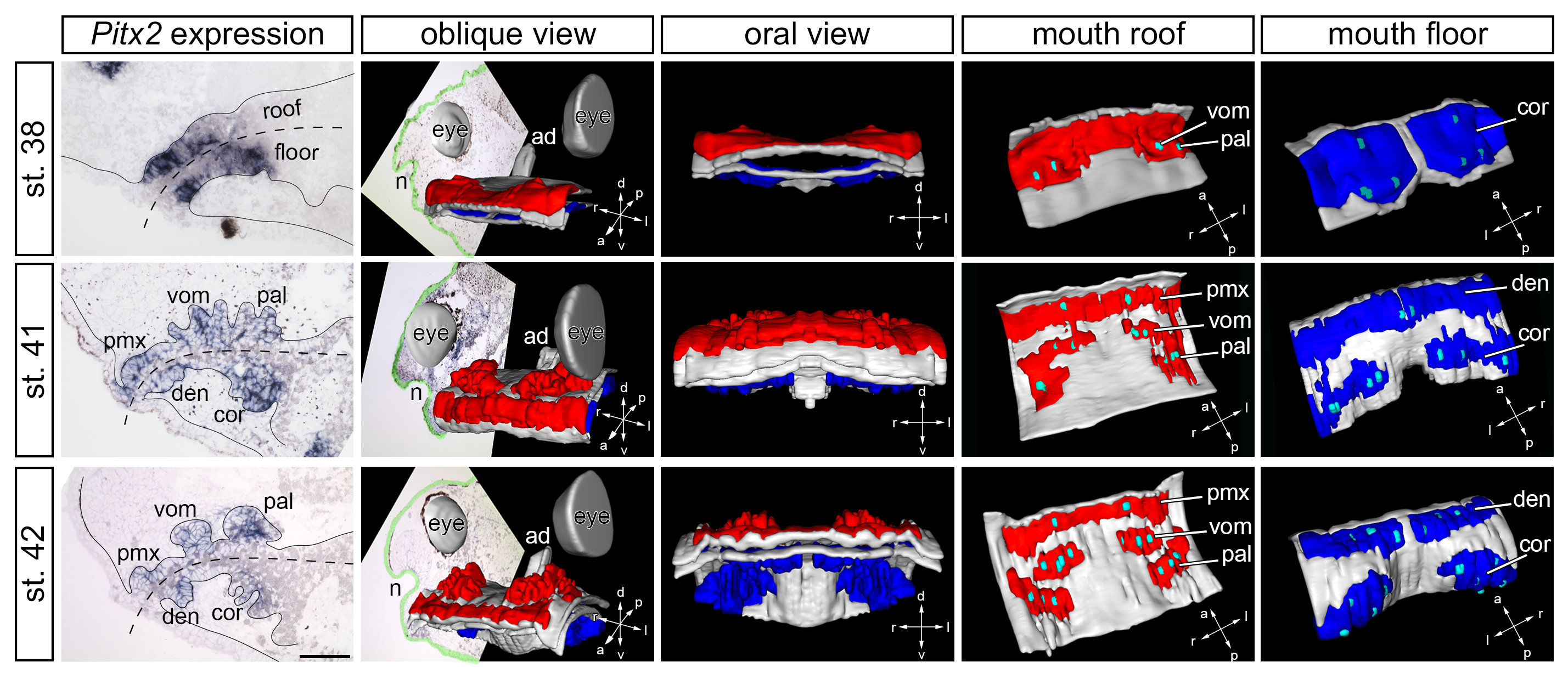

Supplement: Supplementary Figure 2 — 3D models of the developing axolotl dentition based on serial sagittal sections hybridized with the Pitx2 probe. The models visualize the shape and relationship of Pitx2-expressing areas aka tooth competent zones and tooth fields. During embryonic development, the initially compact tooth-competent zones on the mouth roof and floor (st. 38) become compartmentalized into individual tooth fields. The models were reconstructed based on the complete series of sagittal sections hybridized with a Pitx2 probe (left column, solid lines mark the basement membrane of the oropharyngeal epithelia, broken lines mark the place of future oropharyngeal opening). Mouth roof expression is in red color, mouth floor expression in blue color, basal epithelial layer of the oral epithelium in gray color and cyan dots demark positions of tooth germs. ad, adenohypophysis; cor, coronoid field; den, dentary field; n, nasal cavity; pal, palatine field; pmx, premaxillary field; vom, vomerine field. Scale bar equals 100 μm. [file Image_2.TIF]
